# The relationship between sleep disturbance and aggressive behaviour among community-dwelling schizophrenia patients: a moderated mesomeric effect model

**DOI:** 10.1186/s12889-024-19090-9

**Published:** 2024-06-15

**Authors:** Zixiang Ye, Dongmei Wu, Yuchuan Yue, Tao Li, Li Sun, Pei Yu, Yuhao Tong, Li Xiao

**Affiliations:** 1 Department of Nursing, The Clinical Hospital of Chengdu Brain Science Institute, MOE Key Laboratory for Neuroinformation, University of Electronic Science and Technology of China, Chengdu, China; 2 Department of Nursing, Pengzhou Fourth People’s Hospital, Pengzhou, China; 3 Operating room, Clinical Medical College & Affiliated Hospital of Chengdu University, Chengdu, China; 4 Elderly Cardiovascular Care Unit III, Sichuan Academy of Medical Sciences, Sichuan Provincial People’s Hospital, Chengdu, China; 5 Administration Office, The Clinical Hospital of Chengdu Brain Science Institute, MOE Key Laboratory for Neuroinformation, University of Electronic Science and Technology of China, Chengdu, China

**Keywords:** Schizophrenia, Sleep disturbance, Aggressive behaviour, Depressed

## Abstract

**Objective:**

Sleep disturbance is the most common concern of patients with schizophrenia and can lead to a poor prognosis, a low survival rate and aggressive behaviour, posing a significant threat to social security and stability. The aim of this study was to explore the mediating role of depression in the relationship between sleep disturbance and aggressive behaviour in people with schizophrenia living in the community, as well as the regulatory role of family intimacy and adaptability. These findings, in turn, may provide a theoretical basis and constructive suggestions for addressing the physical and mental health problems of these patients.

**Method:**

From September 2020 to August 2021, a convenience sampling method was used to select schizophrenia patients from the community attending follow-up appointments at the Fourth People’s Hospital of Pengzhou City, China. The researchers conducted a survey in the form of a star questionnaire. The survey included questions about general demographic data and disease-related questionnaires: the Pittsburgh Sleep Quality Index (PSQI), the revised Chinese version of the Modified Over Aggression Scale (MOAS), the Self-Rating Depression Scale (SDS), and the Family Adaptability and Cohesion Scale, Second Edition. FACES-II and SPSS 21.0 were used to organize and analyse the data.

**Results:**

A total of 818 schizophrenia patients living in the community participated in the survey, and 785 valid questionnaires were ultimately collected, for a response rate of 95.97%. The results of multivariate analysis indicated that sex, number of psychiatric medications used, outpatient follow-up, history of hospitalization for mental disorders and sleep disturbances were factors influencing aggressive behaviour. Depression played a partial mediating role between sleep disturbance and aggressive behaviour, and the indirect effect size was 0.043 (57.33% of the total). In addition to sleep disturbance, family intimacy (β=-0.009, *P* < 0.01) and adaptability (β=-0.145, *P* < 0.001) can significantly predict depression.

**Conclusion:**

The findings indicate that sleep disturbance in schizophrenia patients in the community is a risk factor for aggressive behaviour, and depression plays a partial mediating role in the relationship among sleep disturbance, aggressive behaviour and family intimacy. In addition, adaptability plays a regulatory role in the relationship between depression and sleep disturbance.

## Introduction

Schizophrenia (SZ) is a common psychiatric disorder characterized by cognitive, emotional, and behavioural disorders [1]. World Health Organization (WHO) survey results demonstrate that the global incidence of schizophrenia is 0.6–1.0%, and schizophrenia patients account for approximately 50% of all individuals with a mental illness [2, 3]. As of the end of 2016, there were approximately 5 million nonhospitalized schizophrenia patients in China, accounting for 90% of the total number of schizophrenic patients [4]. The clinical manifestations of schizophrenia include behavioural disorders, cognitive disorders, delusions, fantasies, and sleep disorders. Among all community-dwelling patients with schizophrenia, approximately 10% exhibit aggressive behaviour [5]. However, the incidence of aggressive behaviour in Chinese patients with schizophrenia is higher (40.2%) [6]. Relevant studies have demonstrated that the repeated cycles of the disease and the continuous deterioration of the condition can lead to a 20-fold greater likelihood of individuals with schizophrenia committing murder compared with the general population [7], and community schizophrenia patients are more likely to engage in aggressive behaviour and pose a greater threat to society than are hospitalized patients [8], representing a serious burden to society and families [9, 10]. Therefore, the prevention and treatment of aggressive behaviour among community-dwelling patients with schizophrenia have become key concerns in social policies and health care.

Sleep disturbance can lead to adverse emotional reactions in patients with schizophrenia. In severe cases, sleep disturbance can trigger self-harm behaviour, aggressive behaviour, and suicide [11–13]. Using a meta-analysis exploring the relationship between sleep and aggressive behaviour, Demichelis OP [14] reported that sleep deprivation in the general population is associated with increased levels of aggression. Another meta-analysis suggested that individuals with schizophrenia have poorer sleep quality and greater levels of aggression [15]. In summary, conducting research on sleep quality and aggressive behaviour in community-dwelling patients with schizophrenia has important public health implications for preventing aggressive behaviour. Therefore, the aim of this study was to test our hypothesis that sleep disturbance is a risk factor for aggressive behaviour in individuals with schizophrenia.

Schizophrenia can trigger various negative psychological conditions, among which depression is the most common. According to reports, approximately 30–70% of schizophrenia patients experience depression. Depression seriously affects patients’ physical and mental health and promotes the deterioration of their condition [16, 17]. Numerous studies have demonstrated that insomnia is an independent risk factor for depression and that sleep disorders in patients with severe mental disorders are positively correlated with depressive episodes [18–23].

People often focus on the self-harm and suicidal tendencies and behaviours of depressed patients and overlook their violent aggressive behaviour. However, clinical studies have demonstrated [24] that depressed patients are three times more likely to commit violent crimes than the general population, and suicidal behaviour also increases the risk of attacking others [25, 26]. Glaser (1967) [27] and Lesse (1974) [28] proposed a performance model through clinical research, suggesting that emotional issues such as depression can alter an individual’s performance behaviour, often leading to anger and expression in behaviour, resulting in aggressive behaviour. Therefore, the aim of this study was to test our hypothesis that depression plays a mediating role between sleep disturbance and aggressive behaviour.

The family plays a crucial role in the healthy development of individual psychology [29]. At present, home-based care is the primary approach for patients with mental disorders in China [30]. Olson et al.’s arched pole model suggests that family functioning is concentrated on two aspects: family intimacy and family adaptability [31]. Family intimacy refers to the emotional connections between family members, whereas family adaptability refers to the degree to which a family system can flexibly handle problems in different stages of family environment development [32]. According to ecosystem theory, the full utilization of family functions has a significant impact on the development of individual psychological characteristics, which contributes to the physical and mental health development of individuals and the cultivation of healthy personalities [33]. Previous studies have reported a negative correlation between family functioning and the degree of depression among family members [34–36]. In addition, family functioning is negatively correlated with aggressive behaviour among family members [37, 38]. Therefore, this study was conducted to test our hypothesis that family intimacy and adaptability play a regulatory role in the relationship between sleep disturbance and aggressive behaviour.

**Fig. 1:**
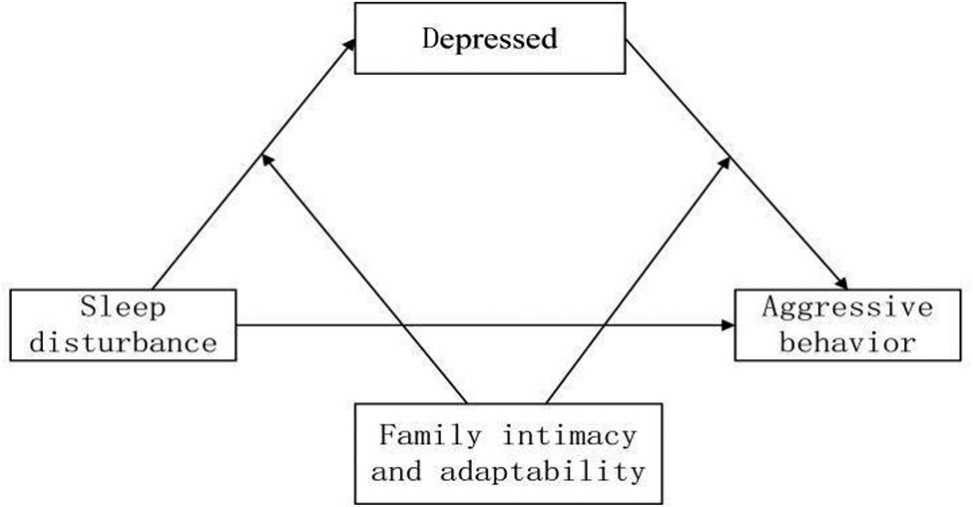
Regulatory mediation model for the relationship between sleep disturbance and aggressive behaviour

At present, although many studies have confirmed the relationship between sleep quality and aggressive behaviour in patients with schizophrenia, there is relatively little research on community-dwelling patients with schizophrenia. In addition, there are currently no relevant research reports on the relationships among sleep disturbance, aggressive behaviour, and depression in patients with schizophrenia. Moreover, sleep disturbance can lead to aggressive behaviour through different pathways, and psychological issues in individuals with schizophrenia are often important mediating factors. Family functioning may play a regulatory role. Protecting the physical and mental health of individuals with schizophrenia is of great scientific significance for maintaining social security and stability. Therefore, the aim of this study was to explore the impact of sleep disturbance on aggressive behaviour in community-dwelling patients with schizophrenia from a psychological perspective and to analyse the mediating role of depression and the regulatory role of family adaptability and family intimacy to provide empirical support and theoretical guidance for interventions for aggressive behaviour in these patients. Figure 1 illustrates the proposed moderated mediation model.

## Methods

### Design and participants

A convenience sampling method was used to select patients with schizophrenia from 28 communities, including the Tianfu Middle Road Community, Tianfu Middle Road Community, Guangming Community, Jinyang Community, Longtan Community, Linjiang Community, and Qingping Community in Pengzhou, China, from September 2020 to August 2021. The questionnaire was distributed via Questionnaire Star. The inclusion criteria for individuals were as follows: (1) aged ≥ 18 years; (2) diagnosed by two or more psychiatrists and meeting the diagnostic criteria for schizophrenia in the United States Diagnostic and Statistical Manual of Mental Disorders, Fifth Edition (DSM-5) [39]; (3) evaluated by psychiatrists and found to be currently in the remission stage of the disease, with a total score on the positive and negative symptom scales of less than 60 points, and considered in a stable condition and able to cooperate with the study; (4) individuals with normal cognitive function; and (5) voluntarily signed the informed consent form. The exclusion criteria were as follows: (1) other mental or neurological disorders, developmental disorders of the brain or severe trauma, or physical diseases; and (2) a history of drug or alcohol dependence. This study was approved by the Ethics Committee of the Fourth People’s Hospital of Chengdu, with review number [2017], ethical examination number (16) and China Clinical Trial Registration Number ChiCTR1800015219, registration date March 15, 2018.

At the end of the study, all survey question data were exported by the researchers from the Questionnaire Star website. A total of 818 people participated in this study, excluding those who took less than 600 s to answer the questions. Ultimately, 785 valid questionnaires were collected, for an effective response rate of 95.97%.

### Measures

#### General demographic characteristics

The general demographic characteristics reported by participants included sex, BMI, family residence, occupation, family monthly income (yuan), marital status and smoking status.

#### Medication compliance

Medication adherence was assessed using the Morisky self-report adherence questionnaire (MAQ) developed by Morisky in 1986. There are a total of 4 items in this questionnaire, among which subjects with a medication adherence score greater than or equal to 3 are considered to have medication noncompliance [40]. The Cronbach’s alpha coefficient of this scale is 0.740–0.762, indicating good reliability.

#### Sleep

The Pittsburgh Sleep Quality Index (PSQI) [41] was used to assess sleep quality. The scale consists of 18 items, including 3 blank questions, 5 multiple choice questions and 10 self-assessment questions. The total score of the scale is composed of seven factors, including sleep quality, sleep time, falling asleep to time, sleep efficiency, sleep disturbance, hypnotics and daytime dysfunction. The total score of this scale ranges from 0 to 21 points, and the higher the score is, the poorer the sleep quality of the study subjects. A total score greater than 7 on this scale indicates sleep disturbance [42]. The Cronbach’s α coefficient of this scale is 0.703–0.778, with good internal consistency.

#### Aggressive behaviour

The revised Chinese version of the Explicit Offensive Behaviour Scale (MOAS) was used to assess aggressive behaviours. The scale includes four types of attack subscales: verbal attack, assault on property, self-attack, and assault on others. Each subscale is rated on a 5-point Likert scale (0–4), with a higher score indicating a greater number and severity of aggressive behaviours. The Cronbach’s α coefficient is 0.813–0.826 [43].

#### Depressed

The Self Rating Depression Scale (SDS) was used to assess the depression status of the study subjects. This scale is mainly used to measure the self-perception status of the study subjects in the past week and consists of 20 items. Each item adopts a 4-level scoring system, with items 1, 2, 3, 7, 8, 9, 10, 15, and 19 having positive scores. The total score of this scale ranges from 0 to 80 points, with a standard score of < 50 points indicating no depression, a score ranging from 50 to 60 indicating mild depression, a score ranging from 60 to 70 indicating moderate depression, and a score > 70 indicating severe depression [44]. The Cronbach’s α coefficient of the scale is 0.861–0.884.

### Family intimacy and adaptability

The Family Intimacy and Adaptability Scale was developed by Olsen et al. in 1982 and was translated and revised into Chinese by Fei LP et al. [45] in 1991; it includes two parts, Family Intimacy and Family Adaptability, with a total of 30 items. Using a 5-point Likert scale, “not”, “occasionally”, “sometimes”, “often”, and “always” are scored as 1, 2, 3, 4, and 5 points, respectively. The higher the score is, the greater the family intimacy and adaptability. The internal consistency of this scale is measured by Cronbach’s α, which ranges from 0.68 to 0.85.

### Statistical analysis

This study used Epidata 3.1 software to input and organize the data, and SPSS 21.0 and process software were used to analyse and process the data. The descriptive analysis method was used to analyse the general demographic characteristics of the research subjects. Univariate analysis of sleep disturbance and aggressive behaviour in schizophrenia patients was conducted using the *χ*^2^ test, while multivariate analysis of aggressive behaviour in schizophrenia patients was conducted using binary logistic regression analysis. The bootstrap method was used to test the mediating regulatory effect in the study. Differences were considered statistically significant at *p* < 0.05.

## Results

### Basic information and correlation analysis of the research subjects

The study population consisted of 414 males (52.74%) and 371 females (47.26%). There were 132 people with sleep disturbance, accounting for 16.82% of the total surveyed population. The proportion of schizophrenia patients with aggressive behaviour was 13.63%.

A comparison of the incidence of sleep disturbance and aggressive behaviour among populations with different demographic characteristics revealed that the incidence of aggressive behaviour in women was greater than that in men (*P* < 0.05), and there was no difference in the incidence of sleep disorders between men and women (*P* > 0.05). The number of psychiatric medications used within 3 months and the incidence of atypical combined typical antipsychotic attacks in patients with schizophrenia were greater than those in patients who had not used antipsychotic drugs or only used antipsychotic drugs, and the differences were statistically significant (*P* < 0.05). However, the difference in the incidence of sleep disturbance between the two groups was relatively small (*P* > 0.05). The incidence of aggressive behaviour in patients with schizophrenia who underwent psychotherapy within 3 months was greater than that in patients who did not (*P* < 0.05), and there was no difference in the incidence of sleep disturbance between the two groups (*P* > 0.05). The incidence of sleep disturbance was greater in smoking patients than in nonsmokers (*P* < 0.05), and there was no difference in the incidence of aggressive behaviour between the two groups (*P* > 0.05), as shown in Table 1.

### Analysis of medication compliance among community-dwelling schizophrenia patients with aggressive behaviour

Among community-dwelling patients with schizophrenia and a history of psychiatric drug use, all survey subjects experienced noncompliance with medication (5.20 ± 1.90). The medication adherence scores of community schizophrenia patients with aggressive behaviour and those without aggressive behaviour were 7.55 ± 0.80 and 4.26 ± 1.27, respectively, with statistically significant differences (t=-15.306, *P* < 0.001).

### Multivariate analysis of aggressive behaviour in community-dwelling patients with schizophrenia

Using aggression as the dependent variable and the statistically significant variables in the univariate analysis as the independent variables, binary logistic regression analysis was conducted. Stepwise regression was used to screen the independent variables, a model with α = 0.05 was entered, and a model with α = 0.10 was removed. The results showed that sex, total and type of psychiatric medication, number of outpatient follow-up visits, history of hospitalization for mental disorders and sleep disturbance were all influencing factors for the occurrence of aggressive behaviour among the surveyed subjects. The parameter estimates and hypothesis test results of the variables included in the model are detailed in Table 2.

**Table 1:** Aggressive behaviour and sleep disturbance with different demographic characteristics (*N* = 785)

Variable		*n*	Aggressive behaviour			Sleep disturbance		
			*n*(%)	χ^2^	***p***	*n*(%)	χ^2^	***p***
Sex								
	Male	414	40(9.66)	11.720	0.001	66(15.94)	0.478	0.490
	Female	371	67(18.06)			66(17.79)		
BMI (kg/cm^2^)								
	Thin	39	7(17.95)	0.816	0.665	7(17.95)	0.174	0.917
	Normal	329	46(13.98)			57(17.33)		
	Overweight and obesity	317	54(17.03)			68(21.45)		
Place of residence								
	Rural area	669	97(16.95)	3.863	0.145	113(16.89)	5.973	0.05
	Town	86	9(10.47)			9(10.47)		
	City	30	1(3.33)			1(3.33)		
Career								
	No career	555	77(16.11)	5.858	0.053	91(16.40)	0.559	0.756
	Career	93	6(6.45)			66(70.97)		
	Other	137	24(17.52			26(18.98)		
Monthly income (yuan)								
	∼ 1000	290	46(15.86)	5.199	0.158	42(14.48)	1.745	0.627
	1001 ∼ 3000	402	51(12.69)			67(16.67)		
	3001 ∼ 5000	99	9(9.09)			21(21.21)		
	5000∼	14	1(7.14)			2(14.29)		
Marital status								
	Unmarried	258	29(11.24)	5.62	0.229	33(12.79)	7.073	0.132
	Married	374	55(14.71)			73(19.52)		
	Divorced	127	18(14.17)			24(18.90)		
	Bereft of one’s spouse	18	2(11.11)			1(5.56)		
	Other	8	3(37.50)			1(12.50)		
Smoking history								
	No	580	81(13.97)	0.438	0.803	86(14.83)	4.278	0.039
	Yes	205	26(12.68)			46(22.44)		
Use and quantity of psychiatric medication								
	0	653	74(12.78)	84.936	0.000	106(16.23)	26.032	0.760
	1	105	14(13.33)			12(11.43)		
	≥ 2	27	19(70.37)			14(51.85)		
Type of psychiatric medication								
	Atypical	35	6(17.14)	9.861	0.007	2(5.71)	13.377	0.002
	Typical	80	22(27.50)			16(20.00)		
	Atypical + typical	17	10(58.85)			8(47.05)		
History of Psychotherapy								
	No	756	99(13.10)	4.982	0.026	131(14.68)	2.918	0.088
	Yes	29	8(27.59)			1(3.45)		
Modified Electric Convulsive Therapy (MECT) treatment history								
	No	778	106(13.62)	0.000	1.000	131(16.84)	0.000	1.000
	Yes	7	1(14.29)			1(14.29)		
Outpatient follow-up frequency								
	More than usual	25	7(28.00)	35.6	0.000	5(20.00)	0.226	0.893
	Less than usual	227	54(23.79)			37(16.30)		
	As usual	533	46(8.63)			90(16.89)		
History of hospitalization for mental disorders								
	No	733	95(12.96)	4.221	0.04	127(17.32)	2.064	0.151
	Yes	52	12(23.08)			5(9.62)		

**Table 2:** Multivariate analysis of factors influencing aggressive behaviour in patients with schizophrenia living in the community

Variable		B	S.E.	Wald	*p*	OR	OR 95% CI	
							Lower limit	Upper limit
Sex	Male							
	Female	0.500	0.024	484.893	0.000	1.651	1.584	1.720
Number of psychiatric medications used	0							
	1	-0.024	0.032	0.347	0.552	0.984	0.926	1.041
	≥ 2	0.121	0.044	9.582	0.000	1.124	1.046	1.217
Outpatient follow-up frequency	Less than usual							
	As usual	-0.642	0.325	5.571	0.042	0.664	0.449	0.951
	More than usual	-0.688	0.376	6.012	0.033	0.597	0.423	0.899
History of hospitalization for mental disorders	No							
	Yes	1.084	0.349	10.102	0.003	3.214	1.427	6.562
sleep disturbance	No							
	Yes	0.726	0.238	12.285	0.000	2.412	1.414	3.328
Constant term		-3.996	0.711	25.458	0.000	0.014	-	-

Note: Binary variable assignment, sex: male = 1, female = 2: types of psychiatric medications used: none = 0, 1 type = 1, 2 or more = 2; outpatient follow-up frequency: 1 = more than usual, 2 = consistent with normal time, 3 = less than usual; history of psychiatric hospitalization: no = 0, yes = 1; sleep disturbance: no = 0, yes = 1

### Mesomeric effect of depression on the relationship between sleep disturbance and aggressive behaviour in community-dwelling schizophrenic patients

In this study, we further explored the relationships among sleep disturbance, aggressive behaviour, and depression in patients with schizophrenia living in the community. Using the SPSS 21.0 plugin for mediating effect analysis, sleep disturbance was included as the independent variable, depression was included as the mediating variable, and aggressive behaviour in schizophrenia patients was included as the dependent variable in Model 4. Bootstrapping was used for testing, with 5000 bootstrap samples set to construct the mediating effect model. The results showed a statistically significant association between sleep disturbance, depression, and aggressive behaviour in patients with schizophrenia. See Table 3.

**Table 3:** Regression analysis between the variables

		β	SE	T	*P*	95% confidence interval				
						LLCI	ULCI	***R*** ^***2***^	***F***	***P***
Depression	Constant	1.068	0.132	8.085	0.000	0.809	1.328	0.223	224.741	0.000
	sleep disturbance	0.400	0.027	14.991	0.000	0.348	0.453			
Aggressive behaviour	Constant	0.017	0.061	0.283	0.778	-0.103	0.138	0.045	36.946	0.000
	sleep disturbance	0.075	0.012	6.078	0.000	0.051	0.100			
Aggressive behaviour	Constant	-0.098	0.062	-1.569	0.117	-0.22	0.025	0.096	41.663	0.000
	Depression	0.108	0.016	6.589	0.000	0.076	0.139			
	sleep disturbance	0.032	0.014	2.360	0.019	0.005	0.059			

The analysis results show that the percentile bootstrap confidence interval of indirect effects does not contain 0 (0.014, 0.076), indicating that there is a mesomeric effect. The percentile bootstrap confidence interval of the direct effect does not contain 0 (0.005, 0.059), indicating that there is some mediation between sleep disturbance and aggressive behaviour in patients with schizophrenia, and the mesomeric effect accounts for 57.33%. See Table 4.

**Table 4:** Mesomeric effect test of depression between sleep disturbance and aggressive behaviour in schizophrenic patients

Variable	Effect	BootSE	BootCI Lower limit	BootCI Upper limit	Effect proportion (%)
Total effect	0.075	0.012	0.051	0.010	100
Direct effect	0.032	0.014	0.005	0.059	42.67
Indirect effect	0.043	0.016	0.014	0.076	57.33

Note: BootSE, BootCI lower limit, and BootCI upper limit refer to the standard error, 95% confidence interval lower limit, and 95% confidence interval upper limit of the indirect effects estimated by the deviation corrected percentile Bootstrap method, respectively

### Analysis of the regulatory effects of family intimacy and adaptability on the relationship between sleep disturbances and aggressive behaviour in patients with schizophrenia

**Fig. 2:**
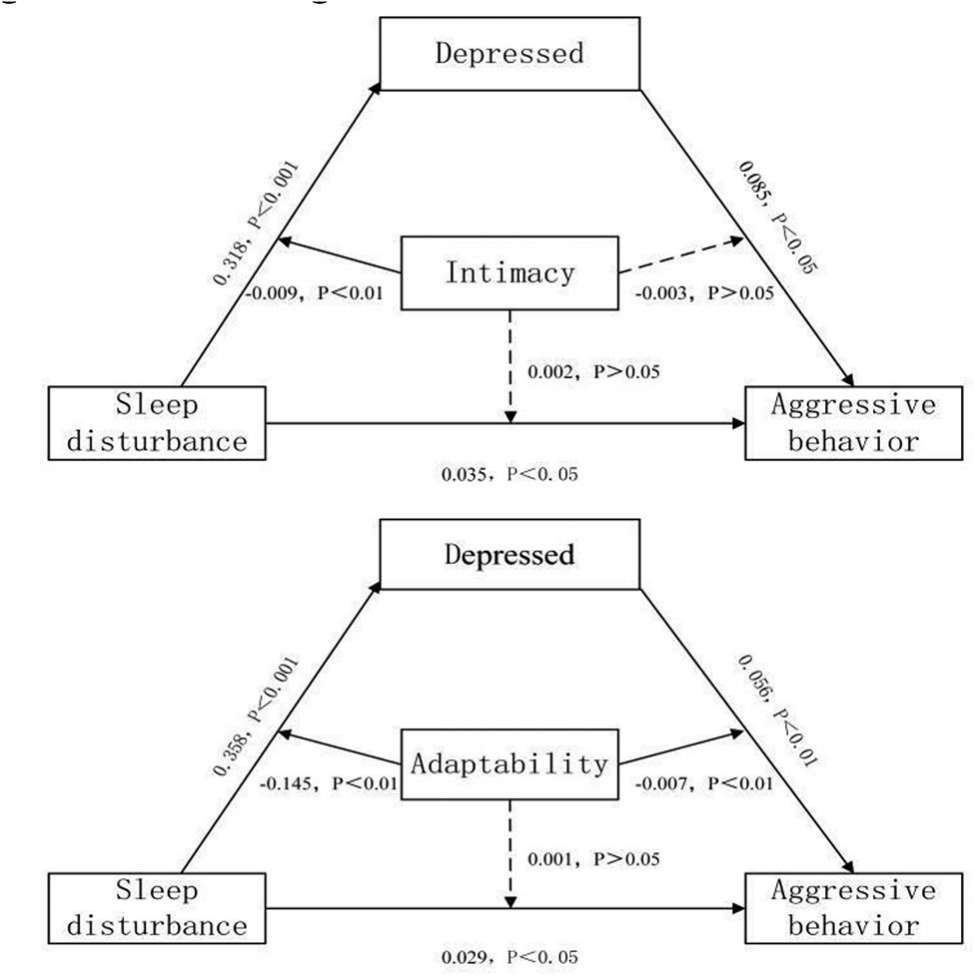
Mediation regulation model diagram

To further explore the role of depression in the relationship between sleep disturbance and aggressive behaviour in patients with schizophrenia living in the community, the plugin process in SPSS 21.0 was used to analyse the mediating regulatory effect. Sleep disturbance was used as the independent variable, depression was used as the mediating variable, family density and total adaptive score were used as the moderating variables, and aggressive behaviour was used as the dependent variable in Model 59. The bootstrap sample size was set to 5000, and a regulated mesomeric effect model was constructed. The results showed that family intimacy and adaptability played a regulatory role in predicting depression through sleep disturbance, that family adaptability also played a regulatory role in predicting aggressive behaviour through depression, and that the results were statistically significant (*P* < 0.05). The mediation effect model is shown in Fig. 2, and the analysis of the mesomeric effect of sleep disturbance on aggression is shown in Table 5.

**Fig. 3:**
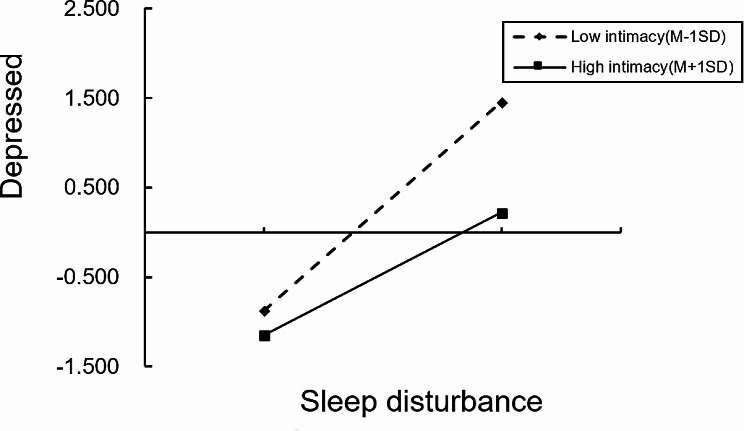
Regulatory effect of family intimacy on the relationship between sleep disturbance and depression

**Fig. 4:**
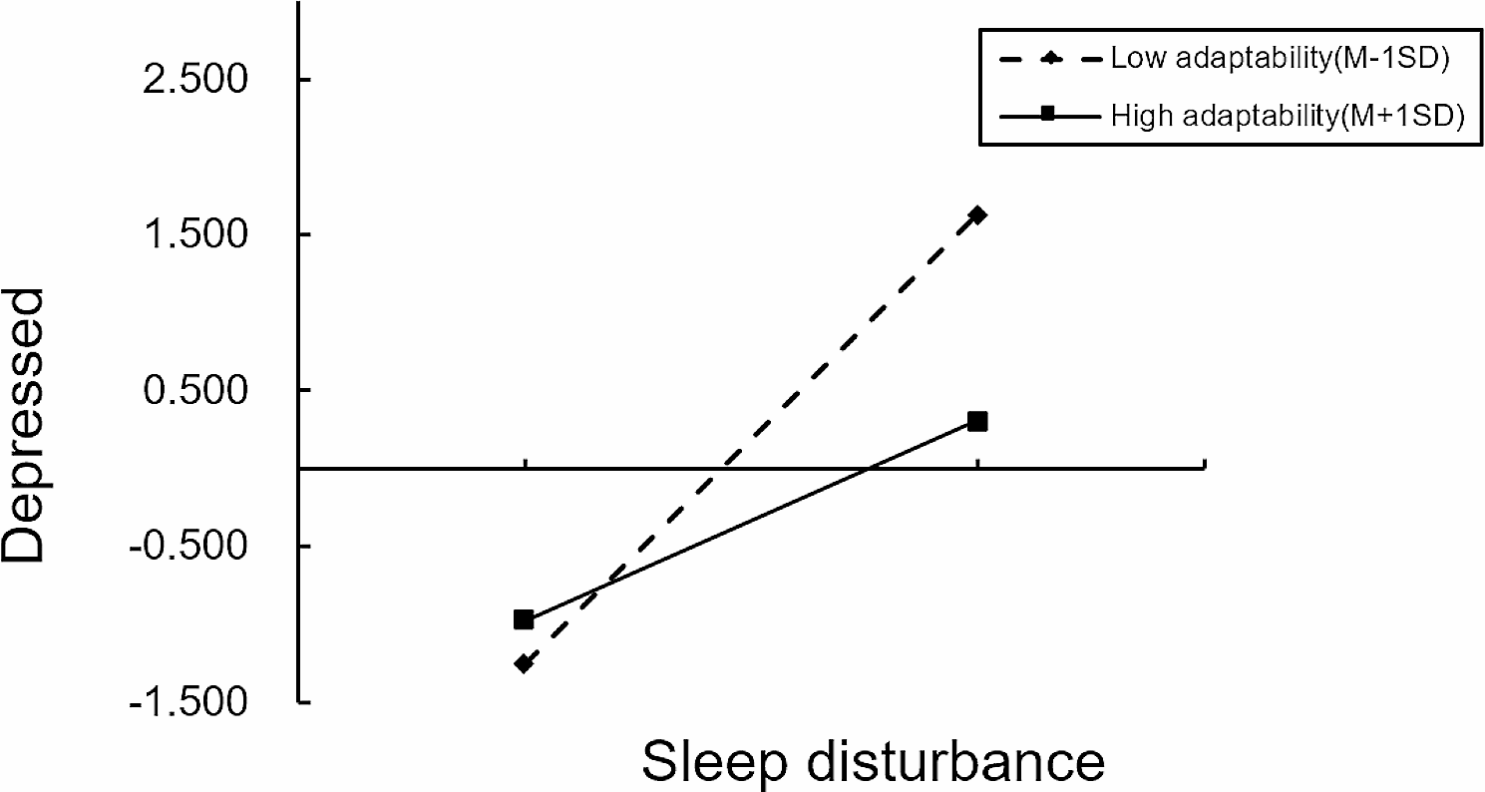
Regulatory effect of family adaptability on the relationship between sleep disturbance and depression

Further simple slope analysis, as shown in Fig. 3, indicated that for patients with lower family intimacy (M-1SD) (such as Z=-1), the upwards trend of depression was significant with increasing sleep disturbance (simple slope = 0.40, t = 13.04, *p* < 0.001). An increase of 1 standard deviation in stress perception led to an increase of 0.40 standard deviations in depression. For patients with high family intimacy (such as Z = 1), the change in depression remained significant as the degree of sleep disturbance increased (simple slope = 0.23, t = 5.34, *p* < 0.001); however, stress perception increased by 1 standard deviation, and depression increased by only 0.23 standard deviations. Figure 4 shows that for patients with lower family intimacy (M-1SD) (such as Z=-1), the increasing trend of depression was significant with increasing sleep disturbance (simple slope = 0.50, t = 14.54, *p* < 0.001); if sleep disturbance increased by 1 standard deviation, depression increased by 0.50 standard deviations. For those patients with high family intimacy (such as Z = 1), as the degree of sleep disturbance increased, the changes in depression were still significant (simple slope = 0.22, t = 5.25, *p* < 0.001), but sleep disturbance increased by 1 standard deviation, while depression increased by only 0.22 standard deviations.

**Table 5:** Analysis of the mesomeric effect of sleep disturbance on aggression

Variable	Aggressive behaviour				Depression			
	***β***	***SE***	***t***	***P***	***β***	***S.E***	***t***	***P***
sleep disturbance	0.035	0.015	2.311	0.021	0.318	0.03	10.679	0.000
Depression	0.085	0.019	4.42	0.000	-	-	-	-
Intimacy	-0.006	0.042	-1.41	0.159	-0.042	0.009	-4.646	0.000
sleep disturbance × Intimacy	0.002	0.001	1.322	0.187	-0.009	0.003	-3.562	0.0004
Depression × Intimacy	-0.003	0.002	-1.96	0.050	-	-	-	-
***R***^**2**^	0.104				0.256			
***F***	18.036				59.667			
***P***	0.000				0.000			
sleep disturbance	0.029	0.014	2.122^*^	0.034	0.358	0.027	13.123^***^	0.000
Depression	0.056	0.016	3.031^**^	0.003	-	-	-	-
Adaptability	-0.015	0.004	-3.909^**^	0.0001	-0.024	0.082	-3.330^**^	0.0009
sleep disturbance × Adaptability	0.001	0.002	-0.889	0.374	-0.0145	0.003	-5.133^***^	0.0000
Depression × Adaptability	-0.007	0.002	-3.988^**^	0.0001	-	-	-	-
***R***^***2***^	0.141				0.256			
***F***	25.480***				59.362***			
***P***	0.000				0.000			

## Discussion

This study presents an investigation of the risk factors for aggressive behaviour in community schizophrenia patients and an analysis of the relationship between sleep disturbance and aggressive behaviour. In addition, this study further examined the mediating effect of sleep disturbance on aggressive behaviour through depression and the moderating effect on the mediating pathway through the interaction term between family functioning and depression. Overall, these findings are consistent with previous studies on the relationships between sleep quality, depression and aggressive behaviour. In addition, depression plays a mediating role in the relationship between sleep disturbance and aggressive behaviour, and family functioning has a significant regulatory effect on the mediating pathway.

The results of this study showed that 132 patients with community schizophrenia had sleep disturbance, accounting for 16.82% of the total survey population, which is consistent with the findings of Robertson I et al. [41]. This study also demonstrated that even if community schizophrenia patients are in a clinically stable period, they can still suffer from sleep disturbance. The results of this study showed that 106 patients with schizophrenia exhibited aggressive behaviour, accounting for 13.63% of the total surveyed population. This result is inconsistent with the findings of studies by Araya T [46] and Zhou JS [47], who reported aggressive behaviour in 26.6% and 35.4% of patients with schizophrenia, respectively. The reasons may be related to the fact that the subjects of this study were mostly community schizophrenia patients in the remission stage who had mild or atypical mental symptoms, and their self-control may have been stronger than that of those in the acute attack stage, reducing the likelihood of aggressive behaviour. Although the incidence of aggressive behaviour among community schizophrenia patients in this study was lower than that among hospitalized schizophrenia patients, aggressive behaviour among community schizophrenia patients cannot be ignored.

The results of the multivariate analysis showed that sleep disturbance in community schizophrenia patients is a risk factor for aggressive behaviour. Studies by Chen ZT [30] and Langsrud K [48] revealed that sleep disturbance in patients with mental disorders is a risk factor for aggressive behaviour, which is consistent with the results of this study. This may be related to the sustained activation of the sympathetic nervous system and the reduced frontal lobe activity caused by sleep disturbance, which can lead to inattention, cognitive impairment, and even behavioural control problems in patients [49]. The results of this study suggest that community workers, medical staff, and family members should be aware of the negative effects of sleep disturbance and aggressive behaviour. At the same time, schizophrenia patients should be encouraged to actively participate in social activities. If schizophrenia patients have poor sleep, the community, medical staff, and family members should engage in more positive psychological communication with these patients to understand the causes of their poor sleep and enable them to seek timely treatment to improve their sleep quality, thereby reducing the occurrence of aggressive and even violent behaviour in community schizophrenia patients, promoting their mental and mental health, reducing the burden of family mental stress, and promoting social harmony and stability.

This study validated the mediating role of depression between sleep disturbance and aggressive behaviour. In the daily life of community schizophrenia patients, sleep disturbance may cause mental health problems, which may lead to the occurrence of depression and subsequently aggressive behaviour. This study revealed that the mediating role of depression is generally consistent with research results on the relationship between past depression and aggressive behaviour [48, 50, 51]. The possible reasons for this may be related to sleep deprivation, decreased activity in the medial prefrontal cortex, and increased activation of the amygdala. The distribution of amygdala activation was consistent with the top-down regulation of emotional responses. When community schizophrenia patients have insufficient sleep, this can lead to the generation of negative emotions. However, in the case of anxiety and depression, the dopamine system at the edge of the midbrain in the human body receives incorrect signals related to a lack of pleasure, causing excessive secretion of dopamine. The release of dopamine is related to hallucinations in patients, leading to aggressive behaviour in schizophrenia patients [52, 53]. Therefore, community healthcare workers need to pay more attention to the mental health status of patients with schizophrenia, especially those with depression, when considering their sleep status. Regular observations of changes in the patient’s condition, actively listening to the demands of schizophrenia patients, providing corresponding mental health services, and reducing the occurrence of depression in community schizophrenia patients are needed to reduce the incidence of aggressive behaviour. In this study, we discuss the potential mediating role that depression plays between sleep disturbance and aggressive behaviour. The indirect impact of depression on aggressive behaviour through sleep disturbance provides insights and directions for further research on the correlation between sleep status and aggressive behaviour in community schizophrenia patients.

As a treatment method for schizophrenia, family psychological education plays an important role in the rehabilitation of community schizophrenia patients [54]. The results of this study show that both family intimacy and family adaptability have a significant interaction with depression, indicating a significant moderating effect on the first half of the mediating pathway, while family adaptability also has a moderating effect on the second half of the mediating pathway. This finding is consistent with the findings of Zahra [55] and Fang H [22], who suggested that higher levels of family intimacy and family adaptability can help individuals avoid aggressive behaviour caused by higher levels of depression. The possible reason for this may be that the family environment indirectly affects symptoms of mental illness through variables such as past mental health [56]. Families with low levels of family intimacy and family adaptability are prone to various conflicts. However, appropriate family intimacy and family adaptability can help promote emotions among family members, create a safe family atmosphere, promote happiness for schizophrenia patients, alleviate depression, and reduce the occurrence of aggressive behaviour disorders [34]. Therefore, while paying attention to patients with schizophrenia, community healthcare workers must also understand their family’s functional status; they can also organize more psychological health promotion activities, regularly hold psychological health lectures, train patients’ family members on their ability to face emergencies, and teach patients methods to relieve psychological stress and communicate with others. At the same time, family members should be guided to provide sufficient care to patients with schizophrenia, understand their true thoughts in a timely manner, and create a warm, safe, and loving family.

### Limitations

The limitations of this study are as follows. First, the use of self-assessment methods for multiple outcome indicators is susceptible to subjective influence and may not accurately reflect the actual situation of the investigators. Future research can use objective measurement methods, such as multichannel sleep monitoring devices or mobile phone trackers, to obtain more accurate data. Second, this study adopted a cross-sectional design, and although it identified risk factors for aggressive behaviour among community schizophrenia patients, there were no causal conclusions. Future research can use a longitudinal design to examine the causal relationships among sleep disturbance, depression, family function, and aggressive behaviour.

## Conclusion

This study confirmed that sleep disturbance in community schizophrenia patients is a risk factor for aggressive behaviour, and mediation effect analysis was used in this study to verify that sleep disturbance has direct and indirect predictive effects on aggressive behaviour and that depression has a direct predictive effect on aggressive behaviour. At the same time, interactions among family intimacy, family adaptability and depression are significant. Therefore, in the future, communities and medical staff should strengthen the evaluation and monitoring of aggressive behaviour and depression in patients with schizophrenia, develop reasonable and effective intervention plans from a positive psychological perspective, and improve patient sleep quality, thereby reducing patient psychological pain and promoting social harmony.

## Data Availability

The datasets used and/or analysed during the current study are available from the corresponding author upon reasonable request.
